# University College, London

**Published:** 1848-06

**Authors:** 


### UNIVERSITY COLLEGE, LONDON.

*Resignation of Mr. Samuel Cooper.*—In a late number we mentioned the appointment of Professor Syme, of Edinburgh, as the successor of the late Mr. Liston, in this metropolitan school. Since then, Mr. Samuel Cooper, author of the Surgical Dictionary, and one of the most eminent surgeons in London, has resigned his professorship of surgery in that institution, after having occupied it with great reputation "for the long space of seventeen years;" not, as he remarks in his "Farewell Address to the Class," entirely from considerations of health, or any inability to continue his lectures, but from disagreement with two of his colleagues, whom he accuses of exercising undue influence in the appointment of Mr. Liston's successor, who was to be associated with Mr. C. in the surgical chair. The announcement by Mr. Cooper of his intention to resign, with the reasons, has given occasion to an unpleasant controversy between him and his colleagues, the more to be regretted from the elevated character of the parties, and the consequent encouragement of a practice much too common among our transatlantic brethren.

